# Anatomical vs. electrophysiological approach for ablation of premature ventricular contractions originating from the left ventricular summit (ISESHIMA-SUMMIT Study)

**DOI:** 10.1093/europace/euae278

**Published:** 2024-11-05

**Authors:** Ryuta Watanabe, Koichi Nagashima, Yasuhiro Shirai, Takayuki Kitai, Takuya Okada, Michifumi Tokuda, Masato Fukunaga, Koumei Onuki, Yosuke Nakatani, Shingo Yoshimura, Seiji Takatsuki, Kenji Hashimoto, Shuhei Yamashita, Masafumi Kato, Fumiya Uchida, Seiji Fukamizu, Rintaro Hojo, Hitoshi Mori, Kazuhisa Matsumoto, Hiroyuki Kato, Kazumasa Suga, Taku Sakurai, Yusuke Sakamoto, Tatsuya Hayashi, Yuji Wakamatsu, Shu Hirata, Moyuru Hirata, Masanaru Sawada, Sayaka Kurokawa, Yasuo Okumura

**Affiliations:** Division of Cardiology, Department of Medicine, Nihon University School of Medicine, 30-1 Ohyaguchi-kamicho, Itabashi-ku, Tokyo 173-8610, Japan; Division of Cardiology, Department of Medicine, Nihon University School of Medicine, 30-1 Ohyaguchi-kamicho, Itabashi-ku, Tokyo 173-8610, Japan; Department of Cardiology, Disaster Medical Center, Tokyo, Japan; Department of Cardiology, Sapporo Cardio Vascular Clinic, Sapporo Heart Center, Sapporo, Japan; Department of Clinical Engineering, Sapporo Cardiovascular Clinic, Sapporo Heart Center, Sapporo, Japan; Department of Cardiology, The Jikei University School of Medicine, Tokyo, Japan; Department of Cardiology, Kokura Memorial Hospital, Kitakyushu, Japan; Department of Cardiology, Kokura Memorial Hospital, Kitakyushu, Japan; Division of Cardiology, Gunma Prefectural Cardiovascular Center, Maebashi, Japan; Division of Cardiology, Gunma Prefectural Cardiovascular Center, Maebashi, Japan; Department of Cardiology, Keio University School of Medicine, Tokyo, Japan; Department of Cardiology, Keio University School of Medicine, Tokyo, Japan; Department of Cardiology, Keio University School of Medicine, Tokyo, Japan; Division of Cardiology, Mie Heart Center, Meiwa, Japan; Division of Cardiology, Mie Heart Center, Meiwa, Japan; Department of Cardiology, Tokyo Metropolitan Hiroo Hospital, Tokyo, Japan; Department of Cardiology, Tokyo Metropolitan Hiroo Hospital, Tokyo, Japan; Department of Cardiology, Saitama Medical University International Medical Center, Saitama, Japan; Department of Cardiology, Saitama Medical University International Medical Center, Saitama, Japan; Division of Cardiology, Japan Community Healthcare Organization Chukyo Hospital, Nagoya, Japan; Division of Cardiology, Japan Community Healthcare Organization Chukyo Hospital, Nagoya, Japan; Division of Cardiology, Japan Community Healthcare Organization Chukyo Hospital, Nagoya, Japan; Department of Cardiology, Tosei General Hospital, Seto, Japan; Division of Cardiovascular Medicine, Saitama Medical Center, Jichi Medical University, Saitama, Japan; Division of Cardiology, Department of Medicine, Nihon University School of Medicine, 30-1 Ohyaguchi-kamicho, Itabashi-ku, Tokyo 173-8610, Japan; Division of Cardiology, Department of Medicine, Nihon University School of Medicine, 30-1 Ohyaguchi-kamicho, Itabashi-ku, Tokyo 173-8610, Japan; Division of Cardiology, Department of Medicine, Nihon University School of Medicine, 30-1 Ohyaguchi-kamicho, Itabashi-ku, Tokyo 173-8610, Japan; Division of Cardiology, Department of Medicine, Nihon University School of Medicine, 30-1 Ohyaguchi-kamicho, Itabashi-ku, Tokyo 173-8610, Japan; Division of Cardiology, Department of Medicine, Nihon University School of Medicine, 30-1 Ohyaguchi-kamicho, Itabashi-ku, Tokyo 173-8610, Japan; Division of Cardiology, Department of Medicine, Nihon University School of Medicine, 30-1 Ohyaguchi-kamicho, Itabashi-ku, Tokyo 173-8610, Japan

**Keywords:** Idiopathic ventricular arrhythmias, Left ventricular summit, Anatomical approach, Electrophysiological approach

## Abstract

**Aims:**

Catheter ablation (CA) of idiopathic ventricular arrhythmias (VAs) from the epicardial left ventricular summit is challenging. The endocardial approach targets two sites: the endocardial closest site (ECS) to the epicardial earliest activation site (epi-EAS) and the endocardial earliest activation site (endo-EAS). We aimed to differentiate between cases where CA at the ECS was effective and where CA at the endo-EAS yielded success.

**Methods and results:**

Fifty-eight patients (47 men; age 60 ± 13 years) were analysed with VAs in which the EAS was observed in the coronary venous system (CVS). Overall, VAs were successfully eliminated in 42 (72%) patients: 8 in the CVS, 8 where the ECS matched with the endo-EAS, 11 at the ECS, and 15 at the endo-EAS. A successful ECS ablation was associated with a shorter epi-EAS–ECS distance (10.2 ± 4.7 vs. 18.8 ± 5.3 mm; *P* < 0.001) and shorter epi-EAS–left main coronary trunk (LMT) ostial distance (20.3 ± 7.6 vs. 30.3 ± 8.4 mm; *P* = 0.005), with optimal cut-off values of ≤12.6 and ≤24.0 mm, respectively. A successful endo-EAS ablation was associated with an earlier electrogram at the endo-EAS [23 (8, 36) vs. 15 (0, 19) ms preceding the QRS; *P* < 0.001] and shorter epi-EAS–endo-EAS interval [6 (1, 8) vs. 22 (12, 25) ms; *P* < 0.001], with optimal cut-off values of ≥18 and ≤9 ms, respectively.

**Conclusion:**

Shorter anatomical distances between the epi-EAS and ECS, and between the epi-EAS and LMT ostium, predict a successful ECS ablation. The prematurity of the endo-EAS electrogram and a shorter interval between the epi-EAS and endo-EAS predicted a successful endo-EAS ablation.

## Graphical Abstract

**Graphical Abstract:**
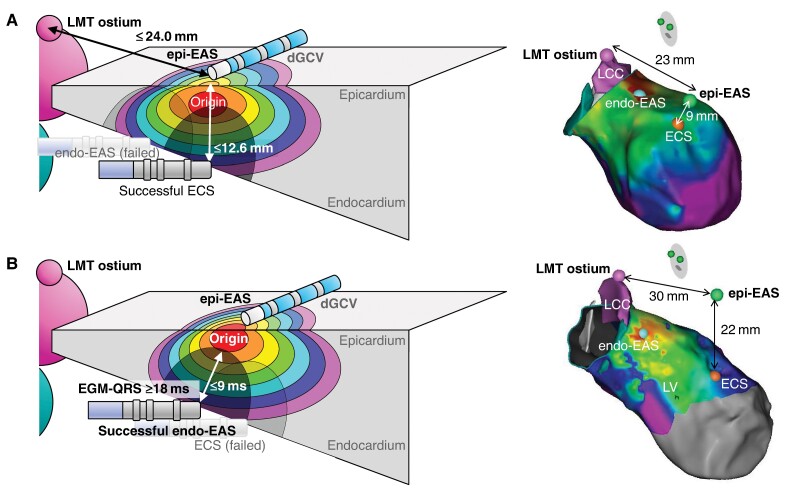
The estimated mechanisms underlying the successful ablation of (*A*) the ECS and (*B*) endo-EAS. The anatomical distances between the epi-EAS and ECS of ≤12.6 mm and between the epi-EAS and LMT ostium of ≤24.0 mm are the determinants of a successful ablation at the ECS. The electrogram at the endo-EAS preceding the QRS by ≥18 ms and the interval of the electrograms between the epi-EAS and endo-EAS of ≤9 ms are the determinants for a successful ablation at the endo-EAS. The details are described in the Discussion. The abbreviations are as shown in *Figure 3*.

**What’s new?**

- The use of a novel multielectrode wire catheter and an intracardiac echocardiography catheter allowed accurate mapping and visualization of the epicardial earliest activation site (epi-EAS) for idiopathic ventricular arrhythmias (VAs) originating from the left ventricular summit (LVS).
- The anatomical distances between the epi-EAS and endocardial closest site (ECS) ≤ 12.6 mm and between the epi-EAS and left main coronary trunk ostium ≤24.0 mm were the determinants for the successful ablation in ECS.
- The electrogram in the endocardial earliest activation site (endo-EAS) preceding the QRS by ≥18 ms and the interval of the electrograms between the epi-EAS and the endo-EAS ≤9 ms were the determinants for the successful ablation in the endo-EAS.
- These findings will contribute to effective treatment strategies for VAs originating from the LVS, where therapy has previously been challenging.

## Introduction

The advancement of catheter ablation technologies and strategies in recent decades has enabled improvements in the ablation outcomes of idiopathic ventricular arrhythmias (VAs) originating from the left ventricular summit (LVS), which is near the coronary venous system (CVS). This includes areas such as the distal great cardiac vein (GCV), anterior interventricular vein (AIV), and communicating veins (CVs).^1–3^ Nonetheless, ablation in this area remains challenging, with an insufficient success rate of ∼50%. The application of radiofrequency (RF) energy is still limited due to its proximity to the coronary arteries, the presence of thick epicardial fat pads, and the constriction of venous vessel diameters, which can hinder the advancement of an ablation catheter.^4–7^ In contrast, alternative endocardial approaches have improved the acute ablation outcomes in specific cases, even when the earliest activation site (EAS) is observed on the epicardium. These endocardial approaches include targeting two sites: one is the endocardial closest site (ECS) to the epicardial EAS (epi-EAS) with a minimal distance^8^ and the other is the EAS on the endocardium (endo-EAS).^1^

Recently, the use of a novel multielectrode wire catheter (EP Star 6F and 2.7Fr EPstar Fix AIV, Japan Lifeline) has been introduced, enabling its placement within the AIV or CV. This advancement has facilitated accurate mapping and visualization of the epi-EAS, revealing that the endocardial site anatomically closest to the epi-EAS and the electrophysiological earliest site in the endocardium are not always identical; in some cases, these two areas are significantly distant from each other. In this context, we conducted a retrospective multicentre study to evaluate the characteristics of VAs originating from the epicardial LVS. We aimed to differentiate between cases where RF at the ECS was effective and where RF at the endo-EAS yielded success. Furthermore, the ultimate goal of this study was to develop a flow chart to guide the selection of the appropriate approach based on both anatomical and electrophysiological data.

## Methods

### Patient characteristics

Between August 2014 and January 2024, data from 58 patients (47 men; age 60 ± 13 years) who underwent catheter ablation of VAs originating from the LVS, including 7 patients with ventricular tachycardias (VTs) and 51 with premature ventricular contractions (PVCs), were analysed. In all patients, the earliest activation was seen on the epicardium within the CVS, specifically in the distal GCV, AIV, or CV. Data from these patients were collected from 12 institutions across Japan for analysis. Among them, one patient had a history of cardiac sarcoidosis. The data collection and analyses received approval from the Nihon University Itabashi Hospital’s review committee and the review committees of each participating centre. Patients consented to the use of their data for this study through an opt-out method.

### Electrophysiological study and mapping

**Figure 1:**
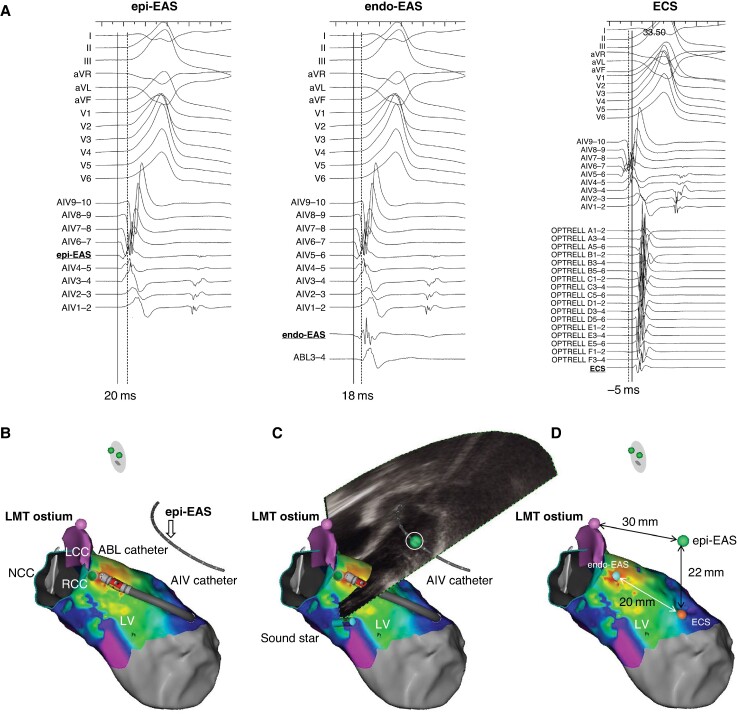
The tagging method of the EAS in the epicardium and LMT using the CARTO system. (*A*) Local electrograms recorded at the epi-EAS, endo-EAS, and ECS. The solid line represents the activation of the local electrogram, and the dotted line indicates the onset of the surface QRS. (*B*) After completing the endocardial activation mapping, the position of the multielectrode wire catheter can be displayed on the CARTO 3 system. A pink tag is also placed on the ostium of the LMT. The white arrow indicates the epi-EAS location seen in AIV5-6. (*C*) The green tag indicates the epi-EAS placed on the CARTO system, utilizing a sound-based geometry, where the fan of the CARTOSOUND is used to traverse the long axis of the wire catheter. (*D*) The orange tag is the ECS of the endocardial site closest to the tagged epi-EAS. The blue tag is the endo-EAS. Abbreviations: ABL, ablation catheter; AIV, anterior interventricular vein; aVF, augmented vector foot; aVL, augmented vector left; aVR, augmented vector rihgt; ECS, endocardial closest site; endo-EAS, endocardial earliest activation site; epi-EAS, epicardial earliest activation site; LCC, left coronary cusp; LMT, left main coronary trunk; LV, left ventricle; NCC, non-coronary cusp; RCC, right coronary cusp.

Using a jugular venous access, a multipolar electrode catheter with an inner lumen (EPstar Fix CS with inner lumen, Japan Lifeline, or RESPONSE 7Fr, Nihon Kohden) was positioned in the distal coronary sinus (CS), and CS venography was performed. Additionally, through the inner lumen, a multielectrode wire catheter (EPstar Fix 2Fr or 2.7Fr EPstar Fix AIV, Japan Lifeline) was inserted into the epi-EAS within the CVS (GCV, AIV, or CVs) under guidance with CS venography. The epi-EAS was the earliest site identified in the entire mapping of the CVS and the right/left ventricular (RV/LV) endocardium. In all patients, electroanatomical mapping was conducted using the CARTO 3 system (Biosense Webster, Diamond Bar, CA, USA). The geometry of the RV and LV, including the aortic cusps, was created with the aid of intracardiac echograms (SOUNDSTAR, Biosense Webster). Activation mapping was then performed in each chamber using a multipolar electrode catheter (DECANAV, PENTARAY, OCTARAY, or OPTRELL, Biosense Webster). Representative local electrograms are presented in *Figure 1A*. Since the activation mapping also captured magnetic matrix information, the position of the multielectrode wire catheter could be displayed on the CARTO 3 system after completing the endocardial activation mapping. A pink tag was also placed on the left main coronary trunk (LMT) ostium using SOUNDSTAR (*Figure 1B*). Subsequently, a green tag was placed at the epi-EAS on the CARTO system utilizing a sound-based geometry where the fan of the CARTOSOUND was used to traverse the long axis of the multielectrode wire catheter (*Figure 1C*). The ECS was defined as the endocardial site closest to the tagged epi-EAS, and the endo-EAS was defined as the EAS on the endocardium (*Figure 1D*). Note that the electrograms at the endo-EAS were always later than those at the epi-EAS in the study patients.

### Ablation

**Figure 2:**
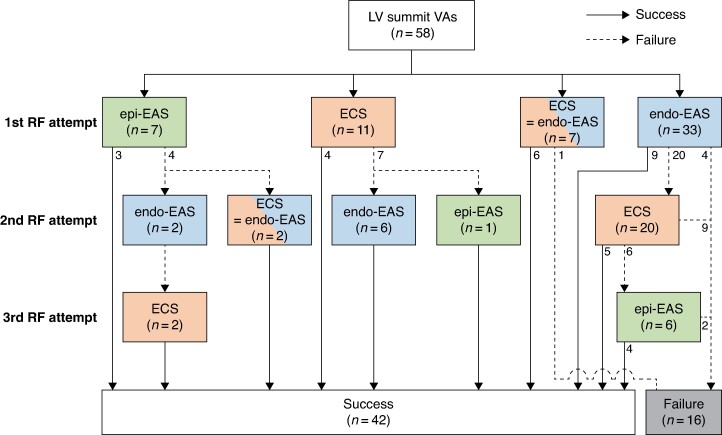
The flow chart of the mapping and ablation procedure. The ablation sites are depicted with consistent colour coding for each tag as used in *Figure 1*. Abbreviations: ECS, endocardial closest site; endo-EAS, endocardial earliest activation site; epi-EAS, epicardial earliest activation site; LV, left ventricle; RF, radiofrequency; VAs, ventricular arrhythmias.

The sequence of the mapping chamber and attempted ablation sites were determined at the operator’s discretion, as shown in *Figure 2*. Coronary angiography was performed if an RF application in the GCV was attempted. RF applications were avoided if the distance from the catheter tip to any major coronary artery was <5 mm. Irrigated RF energy was delivered at a range of 20–25 W inside the GCV, 25–35 W in the sinus of Valsalva, and 30–40 W on the LV endocardium beneath the aortic valve, aiming for an impedance drop of 10–20 Ω. At the LV endocardial sites, the RF applications were typically repeated until unipolar pacing at 10 mA with a 2 ms stimulus strength failed to capture.^1^ Acute success was defined as the absence of any target arrhythmias with/without an isoproterenol administration.

### Follow-up

The patients were monitored for at least 24 h in the hospital before discharge. Post-discharge follow-up included clinic visits with 12-lead electrocardiograms (ECGs) and 24-h Holter monitoring. If patients reported any symptoms, additional 24-h Holter monitoring was performed to identify the cause of the symptoms. Procedural success was defined as either more than an 80% reduction in the VAs on the 24-h Holter monitoring or the complete resolution of the symptoms with no VAs detected on any of the ECGs during the follow-up.

### Statistical analysis

Continuous variables were expressed as the mean ± standard deviation (SD) values or median, and the interquartile ranges are shown in parentheses, as appropriate. A Student’s *t*-test or Mann–Whitney *U* test was used to compare the continuous variables, depending on whether the values were normally distributed, and the χ^2^ test was used to compare dichotomous variables unless the expected values in any cells were <5, in which case, a Fisher’s exact test was used. A *P*-value <0.05 was considered to be statistically significant. Receiver operating characteristic curves were plotted to determine the cut-off values of the ablation-related parameters for an acute successful ablation. Statistical analyses were performed with JMP 16.0 software (SAS Institute, Cary, NC, USA).

## Results

### Electrophysiological characteristics

The patient characteristics and ECG-based features of the VAs are presented in *Table 1*. The QRS morphology in lead V1 was a right bundle branch block (RBBB) pattern in 36 patients (62%) and a left bundle branch block (LBBB) pattern with the precordial transition occurring before V3 in 15 patients (26%) and at or after V3 in 6 patients (10%). All VAs exhibited an inferior axis. The average QRS duration was 158 ± 19 ms. The maximum deflection index (MDI), defined as the interval from the earliest QRS onset to the earliest R wave peak in the precordial leads divided by the QRS duration, was 0.59 ± 0.08. Additionally, 42 patients (72%) had an MDI >0.55.^9^

**Table 1:** Patient characteristics and ECG-based features of VAs

	Patients (*n* = 58)
Age, years	60 ± 13
Men	47 (81%)
LVEF, %	55.6 ± 12.3
Structural heart disease	
IHD	4 (7%)
NICM	6 (10%)
Clinical arrhythmias	
VT	7 (12%)
PVCs	51 (88%)
VAs morphology	
QRS width, ms	158 ± 19
MDI	0.59 ± 0.08
Inferior axis	58 (100%)
Limb leads	
QS pattern in lead I	18 (31%)
R in lead II, mV	1.73 ± 0.43
R in lead III, mV	1.88 ± 0.55
Ratio in III/II	1.09 ± 0.19
R in aVF, mV	1.81 ± 0.45
Q in aVL, mV	1.05 ± 0.39
Q in aVR, mV	0.83 ± 0.22
Ratio in aVL/aVR	1.32 ± 0.52
Precordial leads	
RBBB pattern	36 (62%)
LBBB with R wave transition ≤ V3	6 (10%)
V2 transition ratio	0.63 (0.41, 1.07)

Values are the mean ± SD, median (25th, 75th interquartile range), or *n* (%).

aVF, augmented vector foot; aVL, augmented vector left; aVR, augmented vector rihgt; IHD, ischemic heart disease; LBBB, left bundle branch block; LVEF, left ventricular ejection fraction; MDI, maximum deflection index; NICM, non-ischemic cardiomyopathy; PVCs, premature ventricular contractions; RBBB, right bundle branch block; VAs, ventricular arrhythmias; and VT, ventricular tachycardia

### Radiofrequency application at the earliest activation site on the epicardium and subsequential mapping on the left ventricle endocardium

The epi-EAS was identified within the distal GCV in 17 patients, within the AIV in 40, and within the CV in 1. The electrograms at the epi-EAS preceded the QRS onset by 30 (25, 38) ms. The flow chart of the ablation procedure is illustrated in *Figure 2*. Overall, RF energy was applied within the distal GCV in 14 patients, successfully eliminating VAs in eight patients (57%); successful VA elimination was achieved before the endocardial mapping in three patients (*Figure 2*, upper leftward panel) and after the endocardial mapping in five patients (*Figure 2*, centre and lower rightward panels). An RF application within the CVS was not performed in 44 patients (76%), due to the inability to advance the ablation catheter to the epi-EAS in 32 patients, proximity to the coronary artery in 8, and a high impedance in 4.

Left ventricular endocardial mapping was performed in 55 patients, excluding the three patients in whom a distal GCV ablation was successful before the endocardial mapping. Overall, the locations of the endo-EAS and ECS matched, with a distance of <5 mm in 10 patients, but were ≥5 mm apart from the ECS in 45 patients. In all 45 patients, the endo-EAS was located more towards the valvular direction (towards the aortic valve in 42 and the mitral valve in 3).

### Ablation at the endocardial closest site

Radiofrequency ablation targeted the ECS in a total of 42 patients. Ablation was initially performed at the ECS in 18 patients. Among the remaining 24 patients, the initial ablation was unsuccessful at the endo-EAS in 20, at the epi-EAS in 2, and at both the epi-EAS and endo-EAS in 2. The VAs were successfully eliminated in 19 patients (45%).

**Figure 3:**
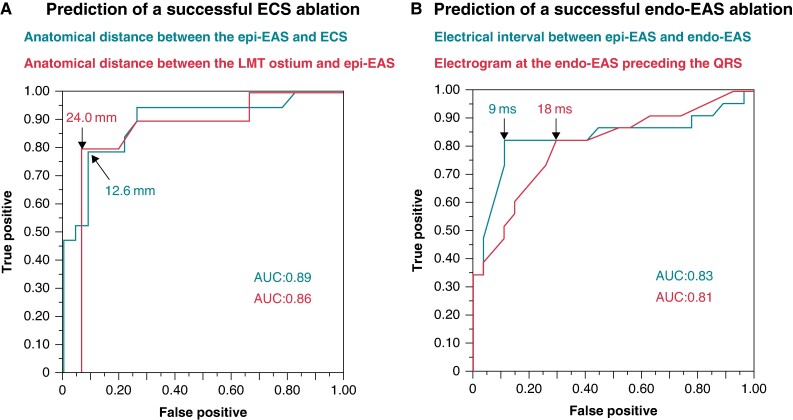
Receiver operating characteristic curves predicting (*A*) successful ablation in the ECS and (*B*) successful ablation in the endo-EAS. Abbreviations: AUC, area under the curve; LMT, left main coronary trunk; the others are as shown in Figure 2.

The anatomical distance between the epi-EAS and ECS was shorter in the patients with a successful ablation at the ECS than in those with a failed ablation (10.2 ± 4.7 vs. 18.8 ± 5.3 mm; *P* < 0.001). Similarly, the anatomical distance between the epi-EAS and LMT ostium was shorter in the patients with a successful ablation at the ECS than in those with a failed ablation (20.3 ± 7.6 vs. 30.3 ± 8.4 mm; *P* = 0.005). The optimal cut-off values for predicting a successful ablation at the ECS were identified as follows: a distance between the epi-EAS and ECS ≤12.6 mm, with an area under the curve (AUC) of 0.89, a sensitivity of 79%, and a specificity of 91%, and a distance between the epi-EAS and LMT ostium of ≤24.0 mm, with an AUC of 0.86, a sensitivity of 80%, and a specificity of 93% (*Figure 3A*). However, there was no difference in the prematurity of the local electrogram at the ECS between the successful ablation at the ECS and the failed ablation [8 (0, 19) vs. 0 (−10, 18) ms preceding the QRS; *P* = 0.173].

### Ablation in the endocardial earliest activation site

Radiofrequency energy was applied at the endo-EAS in a total of 50 patients. Initially, ablation at that site was performed in 40 patients. Among the remaining 10 patients, the initial ablation was unsuccessful at the ECS in six and at the epi-EAS in four. The VAs were successfully eliminated in 23 patients (46%). The local electrogram at the endo-EAS was earlier in the patients with a successful ablation at the endo-EAS than in those with a failed ablation [23 (18, 36) vs. 15 (0, 19) ms preceding the QRS; *P* < 0.001]. However, there was no difference in the prematurity of the electrogram at the epi-EAS [32 (25, 40) vs. 30 (25, 35) ms preceding surface QRS; *P* = 0.367] between these patients. The interval between the electrograms at the epi-EAS and endo-EAS during the VAs was shorter in the patients with a successful ablation at the endo-EAS than in those with a failed ablation [6 (1, 8) vs. 22 (12, 25) ms; *P* < 0.001]. The optimal cut-off values for predicting a successful endo-EAS ablation were identified as follows: an electrogram at the endo-EAS preceding the QRS by ≥18 ms, with an AUC of 0.81, a sensitivity of 83%, and a specificity of 70%, and the interval between the electrograms at the epi-EAS and endo-EAS ≤9 ms, with an AUC of 0.83, a sensitivity of 83%, and a specificity of 89% (*Figure 3B*).

Excluding the eight patients where the endo-EAS and ECS matched, an analysis was conducted on the characteristics of the successful patients at each site separately and included 15 patients with a successful ablation at the endo-EAS and 11 with a successful ablation at the ECS (*Table 2*). The patients with a successful ablation at the endo-EAS were younger than those with a successful ablation at the ECS (55.5 ± 10.6 vs. 67.3 ± 8.9 years; *P* = 0.006). The local electrogram at the epi-EAS tended to be earlier for a successful ablation at the endo-EAS than at the ECS [32 (25, 40) vs. 25 (23, 31) ms preceding QRS; *P* = 0.077].

**Table 2:** Patient characteristics and ECG-related variables in the comparison between the successful ablation in ECS and the successful ablation in endo-EAS

	Successful ablation in ECS	Successful ablation in endo-EAS	*P*-value
	*n* = 11	*n* = 15	
Age, years	67.3 ± 8.9	55.5 ± 10.6	0.006
Men	8 (73%)	13 (87%)	0.620
LVEF, %	56.5 ± 11.7	53.7 ± 16.4	0.637
Structural heart disease	1 (9%)	5 (33%)	0.197
IHD	1 (9%)	2 (13%)	
NICM	0	3 (20%)	
Clinical arrhythmias			0.423
VT	1 (9%)	0	
PVCs	10 (91%)	15 (100%)	
VAs morphology			
QRS width, ms	155.7 ± 17.6	159.5 ± 15.9	0.583
MDI	0.60 ± 0.09	0.55 ± 0.09	0.214
Inferior axis	11 (100%)	15 (100%)	1.000
Limb leads			
QS pattern in lead I	3 (27%)	5 (33%)	1.000
R in lead II, mV	1.69 ± 0.47	1.62 ± 0.39	0.668
R in lead III, mV	1.86 ± 0.60	1.69 ± 0.43	0.452
Ratio in III/II	1.10 ± 0.17	1.06 ± 0.15	0.553
R in aVF, mV	1.76 ± 0.52	1.64 ± 0.38	0.516
Q in aVL, mV	1.04 ± 0.38	0.96 ± 0.30	0.559
Q in aVR, mV	0.80 ± 0.22	0.83 ± 0.21	0.770
Ratio in aVL/aVR	1.33 ± 0.43	1.21 ± 0.41	0.485
Precordial leads			
RBBB pattern	5 (45%)	10 (67%)	0.405
V2 transition ratio	0.71 (0.43, 1.95)	0.57 (0.42, 0.72)	0.454
Mapping (ms)			
Activation time at epi-EAS	25 (23, 31)	32 (25, 40)	0.077
Activation time at endo-EAS	16 (11, 20)	25 (20, 36)	<0.001
Activation time at ECS	4 (−5, 10)	16 (1, 21)	0.111
Interval between (ms)			
Between epi-EAS and endo-EAS	12 (10, 23)	5 (0, 7)	0.001
Between epi-EAS and ECS	24 (17, 31)	22 (12, 32)	0.500
Anatomical distance (mm)			
Between epi-EAS and endo-EAS	19.6 ± 9.9	23.0 ± 8.9	0.384
Between epi-EAS and ECS	10.0 ± 3.5	18.1 ± 6.6	0.001
Between endo-EAS and ECS	11.0 ± 4.3	15.7 ± 9.2	0.215
Between LMT ostium and epi-EAS	20.5 ± 8.6	30.8 ± 9.4	0.039
RF applications			
RF duration at the successful site (s)	72 ± 28	73 ± 20	0.925
Time to VAs elimination (s)	32 ± 24	12 ± 12	0.014
RF duration after VAs elimination (s)	38 ± 31	61 ± 18	0.022

Values are the mean ± SD, median (25th, 75th interquartile range), or *n* (%). *P*-values are for the comparison between the successful ablation in ECS and those in endo-EAS.

EAS, earliest activation site; IHD, ischaemic heart disease; LVEF, left ventricular ejection fraction; MDI, maximum deflection index; NIHD, non-ischaemic heart disease; PVCs, premature ventricular contractions; RBBB, right bundle branch block; RF, radiofrequency; VAs, ventricular arrhythmias; and VT, ventricular tachycardia.

### Radiofrequency duration and the acute ablation outcomes

Among the 58 patients, acute success was achieved in 42 (72%). That included a successful ablation at the epi-EAS in 8 patients, a successful ablation where the ECS matched the endo-EAS in 8, a successful ablation at the ECS in 11, and a successful ablation at the endo-EAS in 15.

In the patients in whom acute success was achieved from the endocardium, the time to the elimination of the VAs was 17 ± 17 s, with the longest time of 60 s at an output of 40 W. The RF duration at the successful ablation site was 72 ± 30 s, with the longest duration of 180 s, resulting in the RF application continuing for 55 ± 30 s after the VAs disappeared. In contrast, in the patients with acute failure, RF was applied for 68 ± 18 s, with the longest application lasting 120 s. The time to the elimination was significantly shorter in the successful endo-EAS ablation group than in the successful ECS ablation group (12 ± 12 vs. 32 ± 24 s, respectively, *P* = 0.014).

### Complications

A complication occurred in one patient, involving a GCV dissection during CS venography, which spontaneously resolved.

### Follow-up

After a median follow-up of 1 (0, 5) month, 41 out of the 58 patients (71%) were free from VA recurrence. Of the 42 patients with an acute success, the VAs recurred in five patients: two with a successful ablation at the ECS, two with a successful ablation at the endo-EAS, and one with a successful ablation at the epi-EAS. Additionally, among the 16 patients with acute ablation failure, 4 experienced a disappearance of the VAs in the chronic phase, while the VAs persisted in 12.

## Discussion

### Main findings

The main findings of this study were as follows: (i) VAs originating from the epicardial LVS were successfully eliminated by RF applications at the epi-EAS inside the distal GCV (*n* = 8), at the ECS remote from the endo-EAS (*n* = 11), at the endo-EAS remote from the ECS (*n* = 15), and at the ECS matching the endo-EAS (*n* = 8); (ii) the anatomical distances between the epi-EAS and ECS of ≤12.6 mm and between the epi-EAS and LMT ostium of ≤24.0 mm were the determinants of a successful ablation at the ECS; and (iii) the electrogram at the endo-EAS preceding the QRS by ≥18 ms and an interval of the electrograms between the epi-EAS and endo-EAS of ≤9 ms were the determinants of a successful ablation at the endo-EAS.

### Acute ablation outcome with the recent anatomical approach

**Figure 4:**
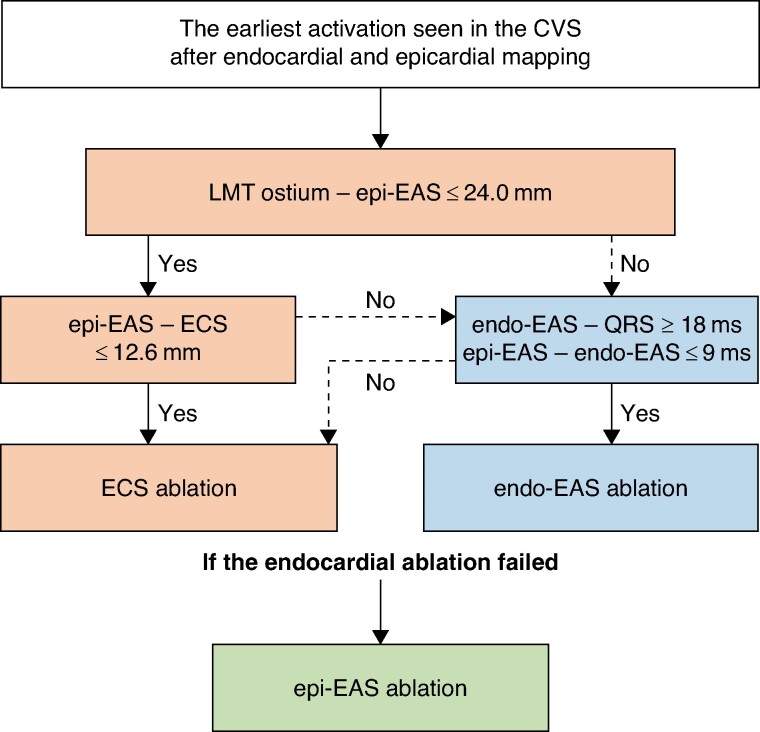
The flow chart of the recommended endocardial ablation strategies for VAs originating from the LVS. Abbreviations: CVS, coronary venous system; the others are as shown in Figure 3.

Compared with the acute successful ablation outcome of 49–53% in previous reports,^1,10^ the acute ablation outcome of 72% in this study appears to be an improvement. This improvement was possibly due to the progression of the alternative endocardial approach because RF applications in the distal GCV are still limited due to the same anatomical barriers such as the proximity of the coronary artery and the constriction of the venous vessel diameter. Although RF energy is recommended to be applied at more than 5–12 mm remote from the coronary arteries to avoid coronary injury,^1,11,12^ a previous report suggests that applying RF energy 10 mm away from the LMT ostium may still pose a risk of chronic coronary stenosis.^13^ Meticulous mapping of the LV endocardium tagging the epi-EAS on the epicardium might play a key role in the successful ablation without any complications such as coronary injury. In the development of an alternative endocardial approach, two targets were considered: the ECS, which targets the anatomically closest site to the epi-EAS, and the endo-EAS, which targets the endocardial EAS during the endocardial mapping. In this study, we elucidated the patient characteristics and electrophysiological features for which each approach was optimal. Based on our findings, we developed a flow chart detailing the recommended ablation strategies for VAs, specifically in patients where the epi-EAS was located in the distal CVS, including locations such as the distal GCV, AIV, or CV after meticulous activation mapping in the CVS and on the LV endocardium (*Figure 4*). Given the favourable acute outcomes of the endocardial ablation strategy and concern about coronary injury during the epi-EAS ablation, we recommended a stepwise approach, prioritizing endocardial ablation targeting the endo-EAS or ECS, while considering an epi-EAS ablation only when necessary. After positioning the multielectrode wire catheter at the EAS within the CVS, the ECS may need to be targeted if the distance from the LMT ostium to the epi-EAS is ≤24.0 mm. Conversely, if this distance is >24.0 mm, targeting the endo-EAS might be necessary. During further endocardial mapping, the RF application should be attempted at the ECS if its distance from the epi-EAS is ≤12.6 mm. On the other hand, if the electrogram at the endo-EAS precedes the QRS by ≥18 ms and the interval between the electrograms from the epi-EAS to the endo-EAS is ≤9 ms, the RF application should be attempted at the endocardial EAS. Combined with previous reports,^1,8,14^ these determinants have become more robust with the accumulation of extensive patient data, thereby strengthening the evidence.

Furthermore, the longer duration of RF applications might play another key role in the improvement in the ablation outcomes as reported in the previous report.^15^ The RF duration at the successful ablation site was 72 ± 30 s, with the longest duration being 180 s, resulting in the RF application continuing for 55 ± 30 s after the VAs disappeared. Based on those results, during endocardial ablation, the RF should be continued for at least ∼60 s after the VA is eliminated, resulting in an RF duration of 90–180 s. Nonetheless, the optimal ablation site should be carefully selected, and redundant RF applications should be avoided, as a prolonged RF application may pose a risk of complications such as a ventricular pseudoaneurysm.^16^

### Potential mechanisms for the successful ablation of the anatomical site and endocardial earliest activation site

The most plausible mechanism underlying the successful ablation at the ECS or endo-EAS is illustrated in the *Graphical Abstract*. With either approach, the VAs could be eliminated if the ablation catheter was positioned close enough to the VA origin for the RF energy to reach. Consequently, for patients who underwent a successful ablation at the ECS, the elimination of the VAs was possible only when the myocardial wall was sufficiently thin. This condition was identified when the anatomical distance between the epi-EAS and ECS was ≤12.6 mm and the distance between the epi-EAS and LMT ostium was ≤24.0 mm (indicating that the successful ablation site was in a periaortic area with thinner myocardium). The local electrogram at the epi-EAS was typically delayed in patients who had a successful ablation at the ECS than in those with a successful ablation at the endo-EAS. Thus, in the group that achieved a successful ablation at the ECS, the origin of the VAs might have been more intramural, that is, relatively closer to the endocardial side, as opposed to the group that achieved a successful ablation at the endo-EAS.

But in patients with a successful ablation at the endo-EAS, the origin of the VAs might be more towards the epicardial side where the RF at the ECS could not reach. From our results, however, the endo-EAS was often remote from the ECS, deviating more towards the valvular region, especially near the aortic valve. That can be explained by the eccentric conduction and thinner myocardial wall in the aortic valvular region.^17,18^ As shown in the *Graphical Abstract*, the myocardial conduction speed may be faster in the long-axis direction than in the short-axis direction. As a result, the endocardial breakout sites for VAs tended to shift towards the direction of the aortic valve, distant from the ECS. In such cases, even if the VA origin was too remote for an effective RF application at the ECS due to the thick myocardium, there remained a slight possibility of eliminating the VA through an RF application from the endo-EAS located in the periaortic area, where the myocardium was thinner. That was indicated by an electrogram at the endo-EAS that preceded the QRS complex by ≥18 ms and an interval between the electrograms at the epi-EAS and the endo-EAS of ≤9 ms. In the current context, VAs not meeting any of the above anatomical and electrophysiological criteria cannot be eliminated by either approach.

### Comparison with alternative ablation technologies

Several alternative ablation technologies have recently been introduced to eliminate VAs originating from the LVS. Ethanol ablation has achieved a successful elimination of LVS-VAs in 68% of RF-refractory cases, increasing to 98% when combined with RF.^19^ However, complications such as a pericardial effusion occur in 5% of cases, requiring percutaneous drainage. Bipolar ablation creates deeper, narrower, and more likely transmural lesions compared with conventional unipolar ablation. A multicentre study of RF-refractory LVS-VAs showed an acute VA elimination in all patients treated with bipolar ablation, with 85% remaining arrhythmia-free after 30 months.^20^ No procedural-related complications occurred. Irrigated needle ablation delivers RF energy deep into the myocardium, with a multicentre study reporting an acute success in 89% of PVC cases and 65% of VT cases, with 78 and 69% remaining arrhythmia-free at 6 months, respectively.^21^ However, pericardial effusion occurred in 3.5% of the cases. Additionally, these technologies are limited to specialized centres. In contrast, ablation with half-normal saline can be performed without special techniques, and it creates larger lesions than with normal saline. However, half-normal saline ablation poses a higher risk of steam pops (12.6%),^22^ and the optimal settings for the RF application with half-normal saline remain unclear. Despite their effectiveness, these new technologies require significant experience, and the risk of serious complications unique to each technology must be accounted for. On the other hand, the alternative endocardial approach has accumulated evidence supporting its efficacy and safety. It is feasible to perform the same procedure without special preparation. Therefore, electrophysiologists may need to initially attempt this approach for complex LVS-VA cases.

### Limitations

Our findings should be interpreted considering the limitations of our study. The first limitation was its nature as a small, retrospective investigation. However, we believe our findings are clinically meaningful, as our sample size of more than 50 patients in this study of VAs was substantial compared with other publications analysing these VAs. Secondly, while it was conceivable that a similar analysis in a much larger patient group might yield different diagnostic cut-off values, we consider this unlikely.

## Conclusions

In 72% of the patients, VAs originating from the epicardial LVS were successfully eliminated by RF applications. The anatomical distances between the epi-EAS and ECS of ≤12.6 mm and between the epi-EAS and LMT ostium of ≤24.0 mm were the determinants for a successful ablation at the ECS. An electrogram at the endo-EAS preceding the QRS by ≥18 ms and the interval of the electrograms between the epi-EAS and endo-EAS of ≤9 ms were the determinants of a successful ablation at the endo-EAS. The RF should be continued for at least ∼60 s after the VA is eliminated, resulting in an RF duration of 90–180 s.

## Data Availability

Data cannot be shared for ethical/privacy reasons.
